# Supplementary material for: The Tumor-Associated Calcium Signal Transducer 2 (TACSTD2) oncogene is upregulated in cystic epithelial cells revealing a potential new target for polycystic kidney disease
Source: PLoS Genet. 2024 Dec 12;20(12):e1011510. doi: 10.1371/journal.pgen.1011510 (PMC11670935; doi:10.1371/journal.pgen.1011510)
Supplement: S1 Fig — (A) Volcano plot showing P10 differentially expressed microRNAs (Omiras). Colored dots indicate FDR <0.05, red are upregulated and blue are downregulated in Experimental vs. Control. Plot was cropped for better visualization of data. (B) Table indicates microRNAs described in (A) that were previously cited in literature with relation to polycystic kidney disease. Citation refers to the individual microRNA or its related family or cluster. Red are upregulated and blue are downregulated in our results. (PDF) [file pgen.1011510.s009.pdf]

Supplemental Figure 1

A

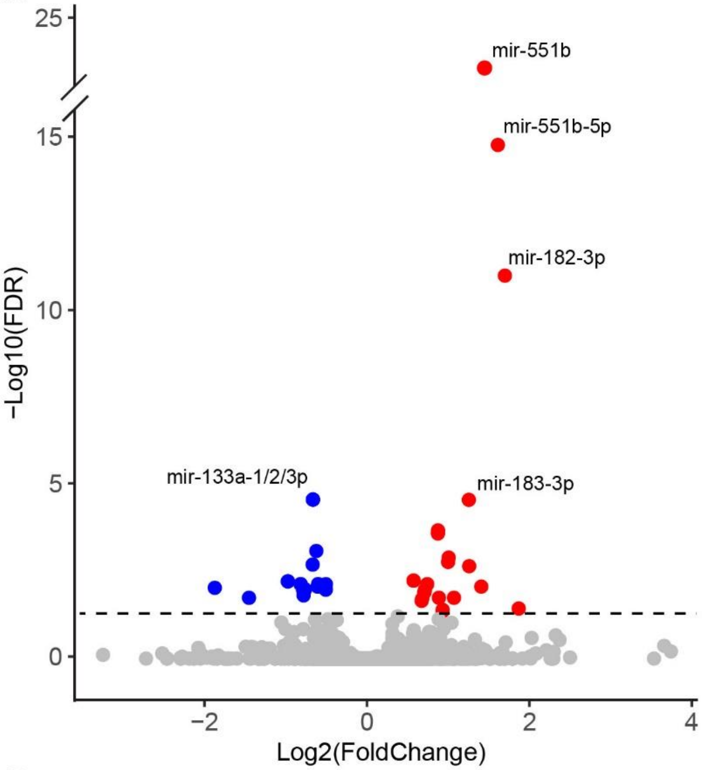

B

miRBase ID	log2 Fold Change	FDR	Gene Family or Cluster	PKD Literature
mmu-miR-18a-3p	0.889	0.018	mir-17-92 cluster	Patel et al (2013)
mmu-miR-92b-3p	0.710	0.012	mir-17-92 cluster	Patel et al (2013)
mmu-mir-92b	0.709	0.012	mir-17-92 cluster	Patel et al (2013)
mmu-miR-182-3p	1.702	<0.001	mir-182 cluster	Pandey et al (2011); Woo et al (2017)
mmu-miR-183-3p	1.258	<0.001	mir-182 cluster	Pandey et al (2011); Woo et al (2017)
mmu-mir-182	1.010	0.001	mir-182 cluster	Pandey et al (2011); Woo et al (2017)
mmu-miR-182-5p	1.002	0.002	mir-182 cluster	Pandey et al (2011); Woo et al (2017)
mmu-mir-183	0.745	0.007	mir-182 cluster	Pandey et al (2011); Woo et al (2017)
mmu-miR-183-5p	0.717	0.010	mir-182 cluster	Pandey et al (2011); Woo et al (2017)
mmu-mir-96	0.683	0.019	mir-182 cluster	Pandey et al (2011); Woo et al (2017)
mmu-miR-96-5p	0.677	0.022	mir-182 cluster	Pandey et al (2011); Woo et al (2017)
mmu-mir-21a	0.935	0.039	mir-21 family	Lakhia et al (2016)
mmu-miR-21a-5p	0.934	0.043	mir-21 family	Lakhia et al (2016)
mmu-miR-222-3p	0.577	0.006	mir-221 cluster	Ben-Dov et al (2014)
mmu-mir-222	0.577	0.006	mir-221 cluster	Ben-Dov et al (2014)
mmu-miR-551b-5p	1.615	<0.001	mir-551 family	Ben-Dov et al (2014)
mmu-mir-551b	1.445	<0.001	mir-551 family	Ben-Dov et al (2014)
mmu-miR-133a-3p	-0.662	<0.001	mir-133 family	Ben-Dov et al (2014)
mmu-mir-133a-1	-0.662	<0.001	mir-133 family	Ben-Dov et al (2014)
mmu-mir-133a-2	-0.662	<0.001	mir-133 family	Ben-Dov et al (2014)
mmu-mir-338	-0.620	0.001	mir-338 family	Ben-Dov et al (2014)
mmu-miR-338-5p	-0.665	0.002	mir-338 family	Ben-Dov et al (2014)
mmu-mir-488	-0.813	0.007	mir-488 family	Pandey et al (2011)
mmu-miR-488-3p	-0.813	0.007	mir-488 family	Pandey et al (2011)

S1 Fig. Small RNAseq of *Pkd2* experimental kidneys vs control at P10 identifies microRNAs with known association in PKD.

(A) Volcano plot showing P10 differentially expressed microRNAs (Omiras). Colored dots indicate FDR <0.05, red are upregulated and blue are downregulated in Experimental vs. Control. Plot was cropped for better visualization of data.

(B) Table indicates microRNAs described in (A) that were previously cited in literature with relation to polycystic kidney disease. Citation refers to the individual microRNA or its related family or cluster: Patel *et al.* 2013 [1], Pandey *et al.* 2011 [2], Woo *et al.* 2017 [3], Lakhia *et al.* 2016 [4], Ben-Dov *et al.* 2014 [5]. Red are upregulated and blue are downregulated in our results.

References

1. Patel V, Williams D, Hajarnis S, Hunter R, Pontoglio M, Somlo S, et al. miR-17~92 miRNA cluster promotes kidney cyst growth in polycystic kidney disease. Proc Natl Acad Sci U S A. 2013;110(26):10765-70. Epub 2013/06/14. doi: 10.1073/pnas.1301693110. PubMed PMID: 23759744; PubMed Central PMCID: PMCPMC3696812.

2. Pandey P, Qin S, Ho J, Zhou J, Kreidberg JA. Systems biology approach to identify transcriptome reprogramming and candidate microRNA targets during the progression of polycystic kidney disease. BMC Syst Biol. 2011;5:56. Epub 2011/04/27. doi: 10.1186/1752-0509-5-56. PubMed PMID: 21518438; PubMed Central PMCID: PMCPMC3111376.

3. Woo YM, Kim DY, Koo NJ, Kim YM, Lee S, Ko JY, et al. Profiling of miRNAs and target genes related to cystogenesis in ADPKD mouse models. Sci Rep. 2017;7(1):14151. Epub 2017/10/28. doi: 10.1038/s41598-017-14083-8. PubMed PMID: 29074972; PubMed Central PMCID: PMCPMC5658336.

4. Lakhia R, Hajarnis S, Williams D, Aboudehen K, Yheskel M, Xing C, et al. MicroRNA-21 Aggravates Cyst Growth in a Model of Polycystic Kidney Disease. J Am Soc Nephrol. 2016;27(8):2319-30. Epub 2015/12/19. doi: 10.1681/ASN.2015060634. PubMed PMID: 26677864; PubMed Central PMCID: PMCPMC4978047.

5. Ben-Dov IZ, Tan YC, Morozov P, Wilson PD, Rennert H, Blumenfeld JD, et al. Urine microRNA as potential biomarkers of autosomal dominant polycystic kidney disease progression: description of miRNA profiles at baseline. PLoS One. 2014;9(1):e86856. Epub 2014/02/04. doi: 10.1371/journal.pone.0086856. PubMed PMID: 24489795; PubMed Central PMCID: PMCPMC3906110.
