# Supplementary material for: The Tumor-Associated Calcium Signal Transducer 2 (TACSTD2) oncogene is upregulated in cystic epithelial cells revealing a potential new target for polycystic kidney disease
Source: PLoS Genet. 2024 Dec 12;20(12):e1011510. doi: 10.1371/journal.pgen.1011510 (PMC11670935; doi:10.1371/journal.pgen.1011510)
Supplement: S2 Fig — (A) Functional enrichment analysis for P6 unique DEGs, P10 unique DEGs, and those common to both lists (CICs). GO, KEGG, Reactome, and WikiPathways databases were probed using g:profiler. Top functions are shown and listed, ranked by p adjusted values. (B) Heatmap comparing differential gene expression for cyst initiation candidates (CICs) in our data (P6, P10) and published pre-cystic mouse data [WP_Pkd2_P7/3/1, WP_Pkd1_P7/3/1 [15], ZS [17], KP2/3/6 [16]. Tacstd2 identified with label and black box. (C) Barplots showing the number of differentially expressed genes (False discovery rate < 0.05) identified in data from the described studies. Our study: P6 and P10 following Pkd2 deletion. Woo et al. [15]: P1, P3, and P7 following Pkd1 or Pkd2 deletion. Kunnen et al. [16]: Weeks 2, 3, and 6 following Pkd1 deletion. Pkd1 models represented by blue bars). Pkd2 models represented by black bars. (PDF) [file pgen.1011510.s010.pdf]

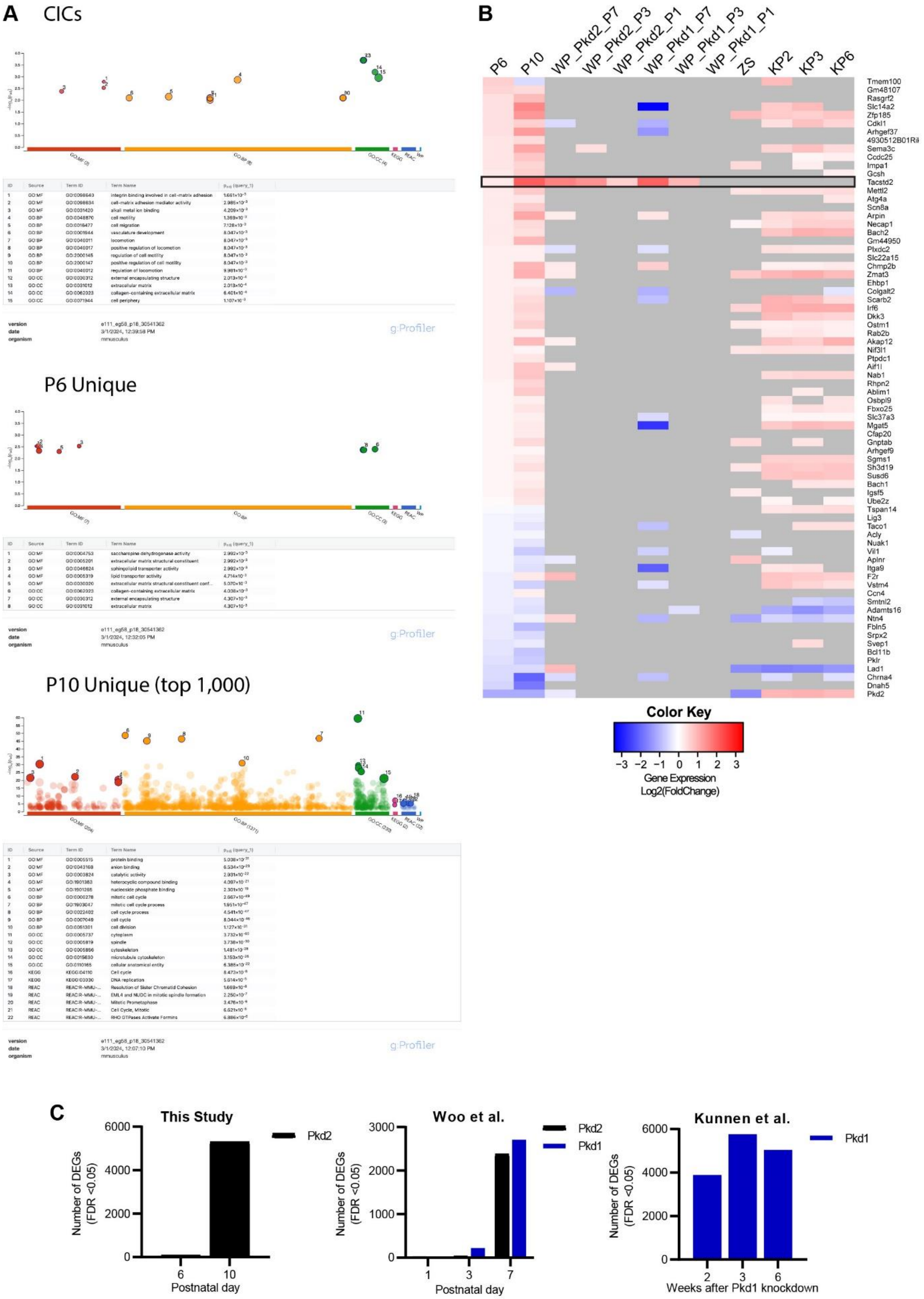
A
CICs
P6 Unique
P10 Unique (top 1,000)
g:Profiler
B
P6
P10
WP_Pkd2_P7
WP_Pkd2_P3
WP_Pkd2_P1
WP_Pkd1_P7
WP_Pkd1_P3
WP_Pkd1_P1
ZS
KP2
KP3
KP6
Tmem100
Gm48107
Rasgrf2
Slc14a2
Zfp185
Cdkl1
Arhgef37
4930512B01Rik
Sema3c
Ccdc25
Impa1
Gcsh
Tacstd2
Mettl2
Atg4a
Scn8a
Arpin
Necap1
Bach2
Gm44950
Plxdc2
Slc22a15
Chmp2b
Zmat3
Ehbp1
Colgalt2
Scarb2
Irf6
Dkk3
Ostm1
Rab2b
Akap12
Nif3l1
Ptpdc1
Aif1l
Nab1
Rhpn2
Ablim1
Osbpl9
Fbxo25
Slc37a3
Mgat5
Cfap20
Gnptab
Arhgef9
Sgms1
Sh3d19
Susd6
Bach1
Igsf5
Ube2z
Tspan14
Lig3
Taco1
Acly
Nuak1
Vil1
Aplnr
Itga9
F2r
Vstm4
Ccn4
Smtnl2
Adamts16
Ntn4
Fbln5
Srpx2
Svep1
Bcl11b
Pklr
Lad1
Chrna4
Dnah5
Pkd2
Color Key
-3 -2 -1 0 1 2 3
Gene Expression
Log2(FoldChange)
C
This Study
Pkd2
Number of DEGs (FDR <0.05)
Postnatal day
Woo et al.
Pkd2
Pkd1
Number of DEGs (FDR <0.05)
Postnatal day
Kunnen et al.
Pkd1
Number of DEGs (FDR <0.05)
Weeks after Pkd1 knockdown

S2 Fig. Differentially expressed mRNAs in pre-cystic and early cystic mouse kidneys.

(A) Functional enrichment analysis for P6 unique DEGs, P10 unique DEGs, and those common to both lists (CICs). GO, KEGG, Reactome, and WikiPathways databases were probed using g:profiler. Top functions are shown and listed, ranked by p adjusted values.

(B) Heatmap comparing differential gene expression for cyst initiation candidates (CICs) in our data (P6, P10) and published pre-cystic mouse data [WP_Pkd2_P7/3/1 [1], WP_Pkd1_P7/3/1 [1], ZS [2], KP2/3/6 [3]. *Tacstd2* identified with label and black box.

(C) Barplots showing the number of differentially expressed genes (False discovery rate < 0.05) identified in data from the described studies. Our study: P6 and P10 following Pkd2 deletion. Woo *et al.* [1]: P1, P3, and P7 following Pkd1 or Pkd2 deletion. Kunnen *et al.*[3]: Weeks 2, 3, and 6 following Pkd1 deletion. Pkd1 models represented by blue bars). Pkd2 models represented by black bars.

References

1. Woo YM, Kim DY, Koo NJ, Kim YM, Lee S, Ko JY, et al. Profiling of miRNAs and target genes related to cystogenesis in ADPKD mouse models. Sci Rep. 2017;7(1):14151. Epub 2017/10/28. doi: 10.1038/s41598-017-14083-8. PubMed PMID: 29074972; PubMed Central PMCID: PMCPMC5658336.

2. Zhang C, Balbo B, Ma M, Zhao J, Tian X, Kluger Y, et al. Cyclin-Dependent Kinase 1 Activity Is a Driver of Cyst Growth in Polycystic Kidney Disease. J Am Soc Nephrol. 2021;32(1):41-51. Epub 2020/10/14. doi: 10.1681/ASN.2020040511. PubMed PMID: 33046531; PubMed Central PMCID: PMCPMC7894654.

3. Kunnen SJ, Malas TB, Formica C, Leonhard WN, t Hoen PAC, Peters DJM. Comparative transcriptomics of shear stress treated Pkd1(-/-) cells and pre-cystic kidneys reveals pathways involved in early polycystic kidney disease. Biomed Pharmacother. 2018;108:1123-34. Epub 2018/10/31. doi: 10.1016/j.biopha.2018.07.178. PubMed PMID: 30372813.
