# Supplementary material for: The Tumor-Associated Calcium Signal Transducer 2 (TACSTD2) oncogene is upregulated in cystic epithelial cells revealing a potential new target for polycystic kidney disease
Source: PLoS Genet. 2024 Dec 12;20(12):e1011510. doi: 10.1371/journal.pgen.1011510 (PMC11670935; doi:10.1371/journal.pgen.1011510)
Supplement: S3 Fig — (A) Dotplot of marker genes supporting cell type assignments in Fig 3E. Markers according to Chen et al. [19]. Dot radius represents percent of cells in cluster expressing the gene. Opacity scale indicates average gene expression level within expressing cells. Row normalized. (B) UMAP from Fig 3E with cell type assignments as labeled by Ransick et al. [21]. (C) Dotplot of marker genes used to annotate S3B Fig. Dot radius represents percent of cells in cluster expressing the gene. Opacity scale indicates average gene expression level within expressing cells. Row normalized. (D) DotPlot visualizing the localization of CICs to the murine kidney cell atlas. Dot radius represents percent of cells in cluster expressing the gene. Opacity scale indicates average gene expression level within expressing cells. Row normalized. Main bars (far left) represent increased expression (red) or decreased expression (blue) in our data; sub bars (left) represent epithelial restricted expression (magenta), broad expression (green), and stromal/indeterminate (gray). Black arrow points to Tacstd2, an epithelial cyst initiating candidate. Blue arrow points to PKD2. (PDF) [file pgen.1011510.s011.pdf]

Supplemental Figure 3

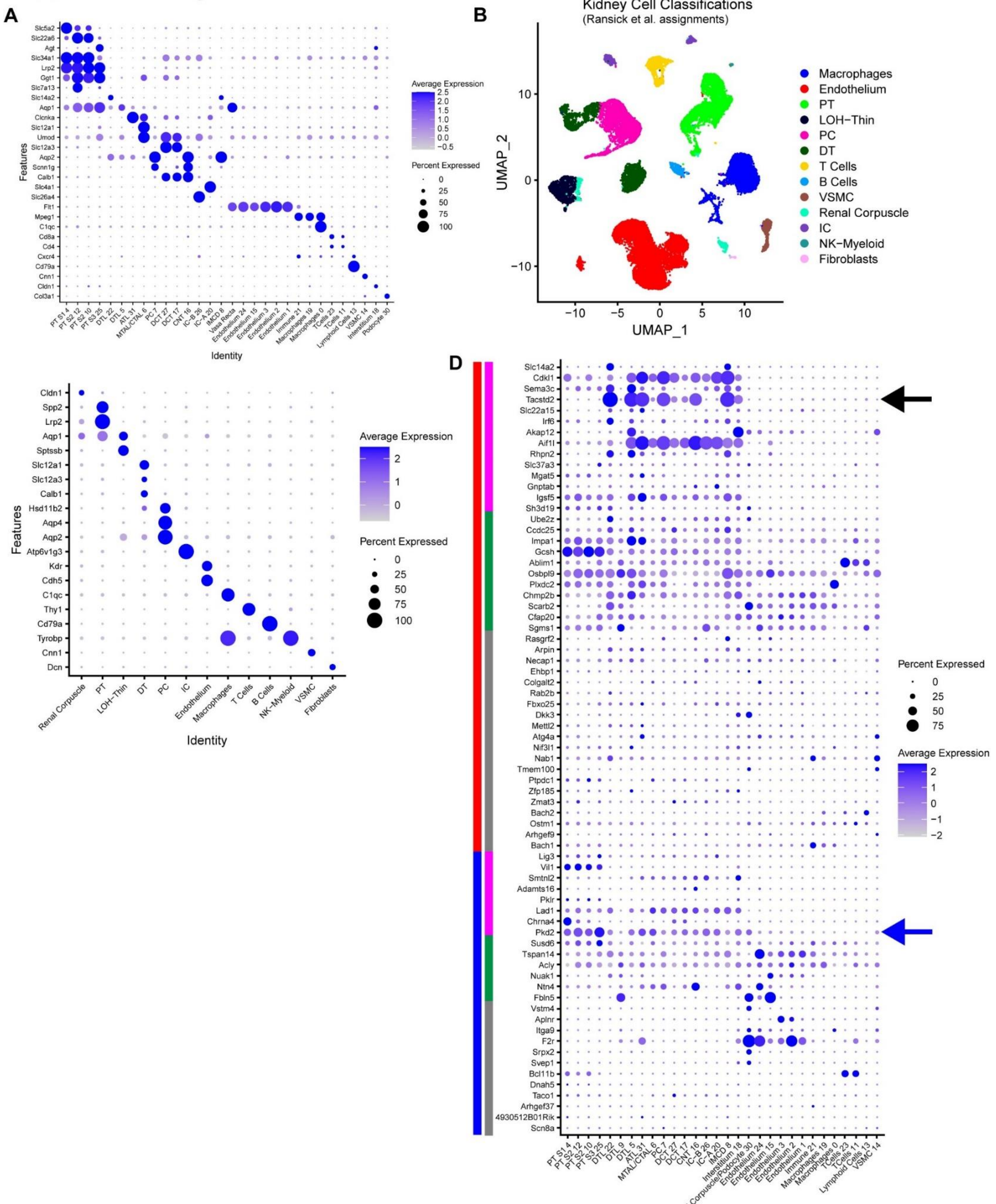

S3 Fig. *Tacstd2* expression in cystic epithelium.

(A) Dotplot of marker genes supporting cell type assignments in Fig 3E. Markers according to Chen *et al.* [1]. Dot radius represents percent of cells in cluster expressing the gene. Opacity scale indicates average gene expression level within expressing cells. Row normalized.

(B) UMAP from Fig 3E with cell type assignments as labeled by Ransick et al. [2].

(C) Dotplot of marker genes used to annotate Fig S3B. Dot radius represents percent of cells in cluster expressing the gene. Opacity scale indicates average gene expression level within expressing cells. Row normalized.

(D) DotPlot visualizing the localization of CICs to the murine kidney cell atlas. Dot radius represents percent of cells in cluster expressing the gene. Opacity scale indicates average gene expression level within expressing cells. Row normalized. Main bars (far left) represent increased expression (red) or decreased expression (blue) in our data; sub bars (left) represent epithelial restricted expression (magenta), broad expression (green), and stromal/indeterminate (gray). Black arrow points to *Tacstd2*, an epithelial cyst initiating candidate. Blue arrow points to PKD2.

References

1. Chen L, Clark JZ, Nelson JW, Kaissling B, Ellison DH, Knepper MA. Renal-Tubule Epithelial Cell Nomenclature for Single-Cell RNA-Sequencing Studies. J Am Soc Nephrol. 2019;30(8):1358-64. Epub 2019/06/30. doi: 10.1681/ASN.2019040415. PubMed PMID: 31253652; PubMed Central PMCID: PMCPMC6683720.

2. Ransick A, Lindström NO, Liu J, Zhu Q, Guo J-J, Alvarado GF, et al. Single-Cell Profiling Reveals Sex, Lineage, and Regional Diversity in the Mouse Kidney. Developmental Cell. 2019;51(3):399-413.e7. doi: 10.1016/j.devcel.2019.10.005.
