# Supplementary material for: The Tumor-Associated Calcium Signal Transducer 2 (TACSTD2) oncogene is upregulated in cystic epithelial cells revealing a potential new target for polycystic kidney disease
Source: PLoS Genet. 2024 Dec 12;20(12):e1011510. doi: 10.1371/journal.pgen.1011510 (PMC11670935; doi:10.1371/journal.pgen.1011510)
Supplement: S4 Fig — (A) All scans used for quantification described in Fig 4. Scale bars 1mm. (PDF) [file pgen.1011510.s012.pdf]

Supplemental Figure 4

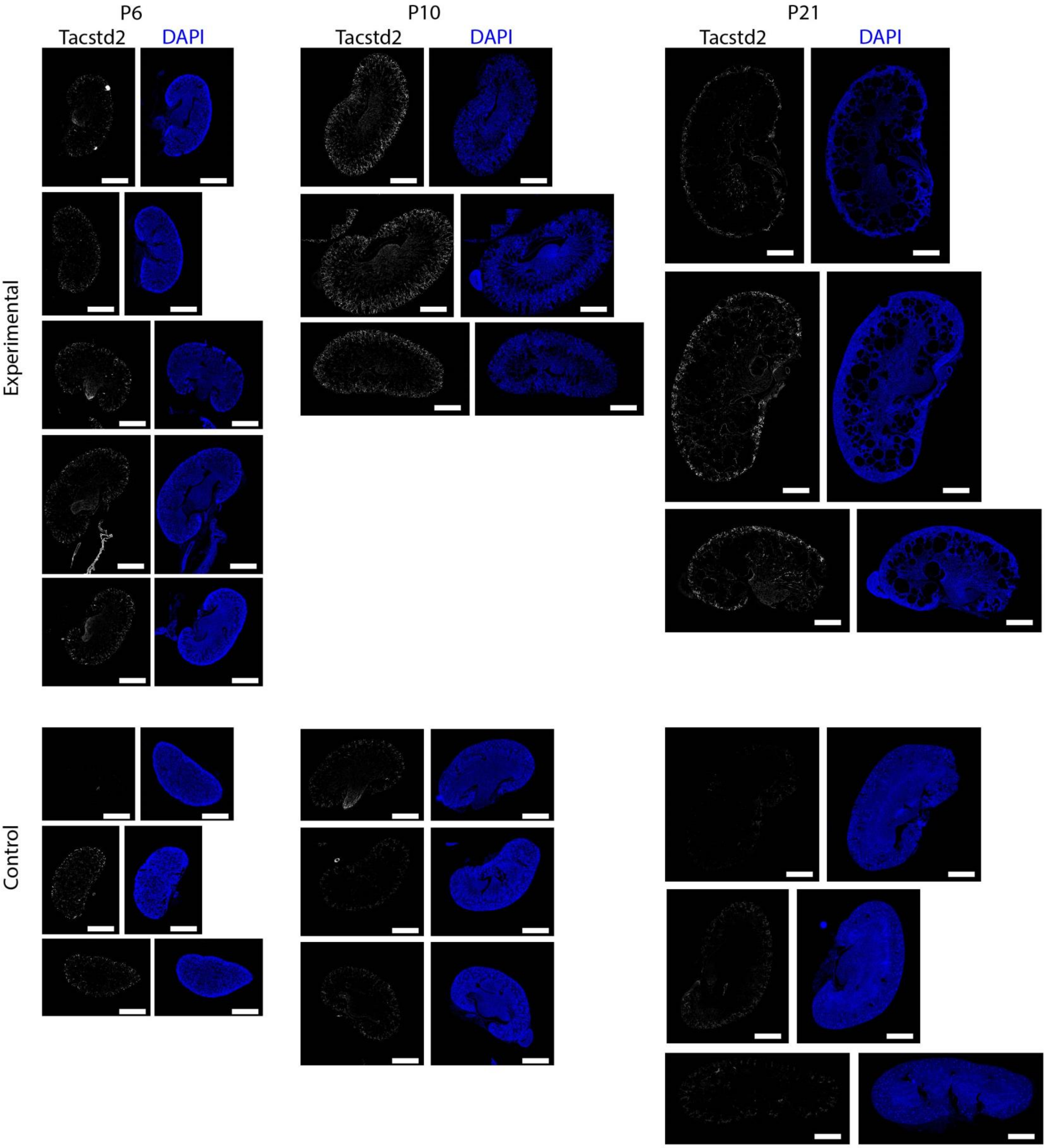

S4 Fig. Tacstd2 expression in cystic mouse renal epithelium.

(A) All scans used for quantification described in Fig 4B. Scale bars 1mm.
