# Supplementary material for: The Tumor-Associated Calcium Signal Transducer 2 (TACSTD2) oncogene is upregulated in cystic epithelial cells revealing a potential new target for polycystic kidney disease
Source: PLoS Genet. 2024 Dec 12;20(12):e1011510. doi: 10.1371/journal.pgen.1011510 (PMC11670935; doi:10.1371/journal.pgen.1011510)
Supplement: S5 Fig — Individual channels of image in Fig 5A. Healthy and ADPKD human kidney sections were probed for TACSTD2 (white), collecting duct marker Aquaporin-2 (AQP2, red) and proximal tubule marker Lotus tetragonolobus Agglutinin (LTA, green). Nuclei were marked by DAPI (blue). Images captured by Zeiss AxioScan.Z1 with 20x objective. Scale bars are 100 microns. (PDF) [file pgen.1011510.s013.pdf]

Supplemental Figure 5

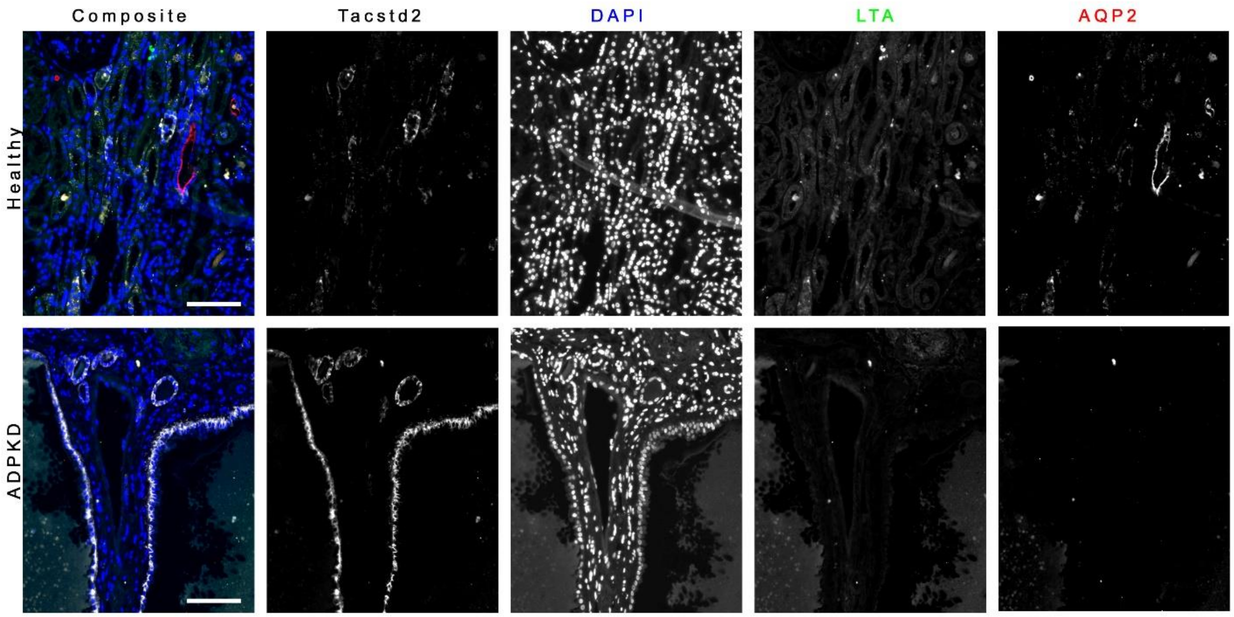


**S5 Fig. Characterizing *TACSTD2* expression in human kidney epithelium.**

Individual channels of image in Fig 5A. Healthy and ADPKD human kidney sections were probed for TACSTD2 (white), collecting duct marker Aquaporin-2 (AQP2, red) and proximal tubule marker Lotus tetragonolobus Agglutinin (LTA, green). Nuclei were marked by DAPI (blue). Images captured by Zeiss AxioScan.Z1 with 20x objective. Scale bars are 100 microns.
