# Supplementary material for: The Tumor-Associated Calcium Signal Transducer 2 (TACSTD2) oncogene is upregulated in cystic epithelial cells revealing a potential new target for polycystic kidney disease
Source: PLoS Genet. 2024 Dec 12;20(12):e1011510. doi: 10.1371/journal.pgen.1011510 (PMC11670935; doi:10.1371/journal.pgen.1011510)
Supplement: Attachment — Submitted filename: PlosGenRebuttal_08_26_2024.docx [file pgen.1011510.s014.docx]

Editor:

The manuscript was fully evaluated at the editorial level and by independent peer reviewers. The reviewers appreciated the attention to an important problem, but raised some substantial concerns about the current manuscript. All reviewers agree about the novelty and significance of this work: TACSTD2 is a novel ADPKD marker that is a potential therapeutic target to prevent cyst formation. There is some disagreement about experimental support and mechanistic understanding for TACSTD2 as a cyst-initiation factor. Authors should address **Reviewer 1s specific comments regarding the site of TACSTD2 expression**. **To address Reviewer 2s serious concerns about lack of experimental support and mechanistic understand, Reviewer 3 points out that it is important to clearly distinguish "coincidence"/"correlation" from "causality".** **Authors may want to include a limitation of study/future directions discussion section to address the concerns of Reviewer 2**. Based on the reviews, we will not be able to accept this version of the manuscript, but we would be willing to review a much-revised version. We cannot, of course, promise publication at that time.

Should you decide to revise the manuscript for further consideration here, your revisions should address the specific points made by each reviewer. We will also require a detailed list of your responses to the review comments and a description of the changes you have made in the manuscript.

**Reviewer #1:**

To find renal cyst initiators, Smith et al. perform transcriptomics on pre-cystic and early-to-moderately cystic kidneys of juvenile Pkd2 conditional ko mice. By comparing with other published ADPKD transcriptomes, including human ADPKD, they identify TACSTD2 as a potential novel drug candidate. They show increased expression of TACSTD2 in tubular epithelia of pre-cystic, moderately and severely mouse cystic kidneys, as well as in cyst-lining cells of human ADPKD kidneys. Using an iPSC-derived organoid model, they also show that loss of PKD1 increases TACSTD2 expression. The manuscript is nicely written and identifies TACSTD2 as a novel ADPKD marker that is aberrantly expressed at early stages of cystogenesis, suggesting this may be a beneficial target to prevent cyst formation.

We thank the reviewer for their kind words and diligent reading of the manuscript. Below we describe how we have addressed their suggestions.

Specific comments:
1. In Fig 4A at P21, whole kidney images appear to show TACSTD2 expression lining cysts that are mostly at the outer edge of the kidney, while cells lining large cysts in the medulla appear to not express TACSTD2. In Fig 4C, please include images of inner cortex as well as of medulla and/or confirm that TACSTD2 is only cortical. If only cortical, how do authors envision TACSTD2 plays a role (or not) in medullary cysts?

The requested images have been added to Figure 4. Tacstd2 is expressed in collecting ducts in the inner cortex and medulla, as well as outer cortex.

2. Fig 4C. The human scRNAseq data show TACSTD2 is also expressed in loop of Henle (Fig 3G). To more fully characterize TACSTD2 expression, this reviewer recommends adding a loop of Henle marker in Fig 4C.

We added images to Figure 4 showing co-stain of Tacstd2 with Aqp1. Aqp1 was chosen as single cell RNAseq showed strong co expression of it with Tacstd2 in descending loops of Henle. Aqp1 is also expressed in proximal tubules and the vasa recta, but these could be distinguished due to the brush border on the proximal tubule cells and the presence of blood cells in the vasa recta.

3. Fig 5A. Similarly, this reviewer recommends including a loop of Henle marker on the human ADPKD sections.

Unfortunately, we have limited access to human samples, and we were unable to make this work.

4. In abstract, please delete final sentence, which is speculative, and because association between ADPKD and cancer incidence is controversial (PMID: 24854270; PMID: 37542051). Alternatively, sentence or line of thought can be included in discussion.

We have clarified that we are discussing TACSTD2 with regard to PKD patients undergoing breast or bladder cancer treatment with TACSTD2 directed therapies. We do not have commentary on kidney cancer and its relation to ADPKD.

“More immediately, our work suggests that PKD patients undergoing TACSTD2-directed treatment for breast and urothelial cancer should be monitored for kidney effects.”

5. P.3, line 73. Italicize genes: PKD1/Pkd1 and PKD2/Pkd2.

In this sentence the abbreviations describe human and murine proteins. Standard nomenclature dictates that human gene names are all capitals whereas mouse genes only have the first letter capitalized and we follow the same format but without italics for proteins.

6. P. 8 line 165, describe the Cre used and timepoint at which deletion of Pkd1/Pkd2 were induced in Kunnen et al. [16] and Zhang et al. [17], similar to how Woo et al. was described.

We have added a detailed description of Kunnen et al. (KspCad-Cre^ERT2^ at 4.5 months) and Zhang et al. (Pax8^rtTA^ TetO-Cre at 7 months) to the main text as well as a reference to Table 2.

7. P. 8, line 179, “human data samples late stage ADPKD”. Please fix phrasing.

We have rewritten the mentioned sentence as “We remain interested in the genes with discrepant expression, as currently available human data necessarily derives from later disease stages than mouse studies, and thus may not fully constitute the milieu of cyst initiation.”

8. P. 9, line 185, change “We” to “we”.

We have altered the capitalization as suggested.

9. P. 12, lines 248-250. Two sentences sound a little contradictory. Recommend adding, “Yet,” or “However,” to beginning of 2nd sentence or rephrasing.

As suggested, we now include an appropriate transition to join the two thoughts.

**Reviewer #2:**

In this study, the authors investigate the role of Tacstd2 in the context of Autosomal Dominant Polycystic Kidney Disease (ADPKD) using an inducible global Pkd2 knockout mouse model. They identify Tacstd2 as significantly upregulated in renal epithelial cells of both mouse PKD kidneys and human PKD tissue biopsies and organoids, but low in healthy renal cells, proposing it as a potential target for PKD treatment. While the study provides valuable insights into expression dynamics of Tacstd2, an interesting oncogene and a novel player in the context of ADPKD, the study is mostly a descriptive study with limited mechanistic evidence supporting the statement that Tacstd2 is indeed a cyst initiating factor. Importantly, no functional experiments were designed to confirm Tacstd2 is required for cystogenesis and that Taxstd2 deficiency ameliorate cystogenesis. Specifically:
1. The use of a global-inducible system (Cagg-CreER) for Pkd2 deletion is considered as a weakness. A segment-specific inducible Cre/ERT2, KspCad or Aqp2 system should be used to minimize noise introduced by ubiquitous Pkd2 deletion across the kidney, potentially may revealing more candidates in pathogenic renal tubules.

We agree with the reviewer that a segment-specific Cre model could potentially reveal different or more candidates. However, this would limit our search to a particular tubule segment, which could be criticized as potentially missing important changes in other segments where cysts could initiate. But more importantly, our experimental approach was successful.

2. The physiology of P6 kidney is very different than adult kidneys in which ADPKD occurs. An adult-inducible PKD rodent model or hylomorphic Pkd1RC/RC line would be better for identifying cyst-initiating factors.

We agree that the P6 kidney is different from an adult kidney. However, the statement implying that ADPKD cysts only develop in adults is incorrect. Numerous studies show that cysts initiate *in utero*. See Pretorius *et al.* 1987 (PMID: 3295293) and Fick *et al.* 1993 (PMID: 8338917) for evidence of *in utero* cysts in ADPKD and Grantham *et al.* 2010 (PMID: 20360307) and De Rechter *et al.* 2017 (PMID: 29326910) for discussion of the importance of *in utero* cysts to disease progression.

It would be difficult to define an early time point in disease progression in the Pkd1^RC/RC^ model, and I doubt that the equivalent of the P6 data set could be obtained with this model. It should be noted that we compared our data set with gene expression datasets from adult-onset models (see Table 2).

3. In P6 kidneys, the study identifies a limited number of differentially expressed genes (DEGs) with subtle fold changes, including Tacstd2 which shows only a modest increase (~25% more than controls). This is consistent with the IF images at P6 (Fig. 4A) that Tacstd2 indeed expresses in healthy kidneys. This challenges the assertion that Tacstd2 acts as a primary cyst-initiating factor.

We acknowledge that Tacstd2 is expressed in healthy mouse and human kidneys (described in lines 207-208 and shown in Fig3 F & G). However, its expression is significantly and consistently higher in cystic tissues. There are many genes for which changes in baseline expression induce pathology. Changes in Tacstd2 expression could be a consequence or cause of cyst growth, and therefore this paper does not claim to prove that Tacstd2 is a primary cyst initiation factor, but rather to identify and characterize it as a possible factor.

4. In vitro and in vivo functional characterizations are needed to validate Tacstd2 as a critical cyst initiating factor, which is central statement of the current study. These experiments would provide mechanistic insights into Tacstd2's role in cystogenesis and clarify whether its deficiency ameliorates cyst formation.

Agreed, and mechanistic experiments are in process. In this paper we limit our claims to the novel description of Tacstd2 expression in healthy and cystic kidney tissues.

5. While the cross-referencing with human ADPKD data in Figure 3 is informative, it should be noted that this data originates from cystic kidneys rather than pre-cystic stages. Thus, validating Tacstd2 as a cyst-initiated factor but not a secondary target during cystogenesis is important.

Agreed, the limitations of access to human ADPKD tissue is acknowledged on line 188 and has been added to the Discussion.

**Reviewer #3:**

In their study "The Tumor-Associated Calcium Signal Transducer 2 (TACSTD2) oncogene is upregulated in pre-cystic epithelial cells revealing a new target for polycystic kidney disease", Smith AO et al. describe an mRNA sequencing-based approach around the timepoint of initial cystogenesis in an inducible Pkd2 knockout mouse model that leads to the identification of Tacstd2.

The authors present a well-conceived and technically well performed investigation addressing a large unmet need in ADPKD therapeutics development; namely the understanding of distinguishing molecular features of early cystic epithelia. The authors should be applauded for this nice study, and it should be considered for publication in PLOS Genetics after minor revision.

The mouse model is well suited to address the experimental questions and the RNA collection, sequencing and data processing and analysis, as well as the comparative analysis with other published data sets is state of the art. The authors then validate the upregulation and specificity of TACSTD2 in cystic epithelia in human ADPKD tissue and in an organic model. I do not have any comments regarding the well conducted technical aspects of the study.

The authors need to be careful about the right language to clearly distinguish "coincidence"/"correlation" from "causality". At various point in the manuscript (for example, p.6 ln.123 "factors that PROMOTE cyst initiation"; p.7 ln.156 "CICs most likely to DRIVE early cystic transformation") there is the risk of overstatement (the authors correct it later in ln.126 "cyst initiation CANDIDATES). The paragraph p.9 ln.200 "Validating Tacstd2 as a cyst initiation candidate" is misleading, because the reader may get the impression that a causative mechanistic role if Tacstd2 in cyst initiation is being validated. Instead, the authors only demonstrate specificity of Tacstd2 as a marker in early cystic epithelia.
It seems important to convey the message that Tacstd2 (and all other DEGs) coincide in their individual expression pattern with the time point of cystogenesis, but that causality can only be ascribed if further experiments prove it necessary for early cyst formation. Obviously, for therapeutic targeting of early cystic epithelia by ADCs, identifying differentially expressed specific surface markers is fully sufficient, regardless of their causative contribution to the cystogenic process.
Please read your own work carefully under that aspect and re-write those passages where the distinction of causality and correlation is not clear.

At beginning of 3^rd^ paragraph of 1^st^ results section, clarified: “The scope of this work was to identify and characterize strong candidates for future mechanistic and possibly therapeutic studies with regards to cyst initiation.”

Deleted “factors that promote”

Reworded “CICs most likely to drive cystic transformation”: “To filter candidate genes by predicted probability of participation in early cystic transformation, we leveraged existing data from the field.”

Replaced “validated” with “characterized” in reference to Tacstd2

Addressed additional areas where causality vs coincidence may have been unclear:

- Abstract: “genes with potential to promote cyst initiation”; “making it an excellent candidate for mechanistic exploration of its role in cyst initiation”

- Intro: deleted “that may drive cyst initiation”; added “as a strong candidate for further investigation”

- Results: Deleted “suggesting that CICs may act broadly to induce cysts”; Replaced “CICs” with “Factors” 206

- Discussion: Added limitations paragraph as noted in response to Reviewer 2
