# Supplementary material for: The Tumor-Associated Calcium Signal Transducer 2 (TACSTD2) oncogene is upregulated in cystic epithelial cells revealing a potential new target for polycystic kidney disease
Source: PLoS Genet. 2024 Dec 12;20(12):e1011510. doi: 10.1371/journal.pgen.1011510 (PMC11670935; doi:10.1371/journal.pgen.1011510)
Supplement: Attachment — Submitted filename: ResponsetoReviewer_10_31_2024.docx [file pgen.1011510.s015.docx]

Reviewer #2: 1. In light of my previous critiques regarding whether TACSTD2 can be classified as a CIC, we should agree that the P6 data is crucial, as P10 kidmeys already show microscopic cysts, especially in renal cortex. However, the observation in P220 that “At P6, the 220 differences did not reach significance (Fig 4C)” renders the conclusion that TACSTD2 is upregulated in pre-cystic stage and potential a CIC invalid.

While protein level as quantified by immunohistochemistry was not statistically significant at P6, the message level was significantly elevated. To address this reviewer’s concerns, we have made the changes to the text as they suggested and are described below.

2. Consequently, the authors should reconsider the title “…upregulated in pre-cystic kidneys…” and the abstract statement “Tumor-associated calcium signal transducer 2 (Tacstd2) stood out as an epithelial-expressed gene with elevated levels prior to cystic transformation that further increased with disease progression”. This is a promising candidate for PKD, but at this stage, without functional characterization, TACSTD2 should primarily be described as a potential key player but not initiator in the early stages of cystogenesis.

The full title now reads: “The Tumor-Associated Calcium Signal Transducer 2 (TACSTD2) oncogene is upregulated in cystic epithelial cells revealing a potential new target for polycystic kidney disease: The new short title reads: “TACSTD2 oncogene with potential cystogenic properties in PKD”. Lastly we have altered the abstract a follows: “*Tumor-associated calcium signal transducer 2* (*Tacstd2*) stood out as an epithelial-expressed gene with elevated levels early in cystic transformation that further increased with disease progression.”

3. In line 280, I suggest changing “To verify that Tacstd2 is a bona fide epithelial cyst initiating candidate…” to “To characterize how Tacstd2 is regulated during early stage of cystogenesis,” since none of the experiments conducted confirm that Tacstd2 is a bona fide CIC.

We changed line 280 to read: “To characterize how Tacstd2 is regulated during early stage of cystogenesis we immunostained tissues for Tacstd2.”

4. Multichannel staining of human patient kidneys in Fig 5 should include individual channels to enhance readers' understanding of TACSTD2 in different renal tubular segments in both healthy and cystic kidneys.

A new supplemental figure S5 has been added with each channel shown individually.
